# A unique ~12 ka subaerial record of rift-transform triple-junction tectonics, NE Iceland

**DOI:** 10.1038/s41598-019-45903-8

**Published:** 2019-07-04

**Authors:** Derek Rust, Malcolm Whitworth

**Affiliations:** School of Earth and Environmental Sciences, University of Portsmouth, Burnaby Road, Portsmouth, PO1 3QL UK

**Keywords:** Natural hazards, Physical oceanography, Tectonics

## Abstract

In northern Iceland the European-North American plate boundary is broad and complex but includes a remarkable subaerial triple-junction intersection between the Husavik-Flatey Fault (HFF) dextral transform and rifting in the Northern Volcanic Zone. Fortuitously, the triple junction occurs in a sheet of ~12 ka pahoehoe lavas; a *tabula rasa* recording innumerable fault features displayed in exquisite detail. High-resolution drone imagery, coupled with 120 field measurements of fault slip directions and opening amounts, made possible the mapping and analysis of this detail and, importantly, enabled recognition and exclusion of potentially misleading primary deformation features associated with emplacement of the lavas. Rift-transform interactions in this natural laboratory have remained spatially stable throughout post-glacial time, although with transform-affinity faults reactivated to accommodate rift extension and transform ‘encroachment’ into the rift domain. First-order en-echelon Riedel fault complexes are recognised, linked by transpressional faulting and compressional strike-slip relay ramps, as well as second-order R shears, R’ and P shears, and previously undescribed R’ Riedel-in-Riedel relationships. A pahoehoe flow front offset along a first-order Riedel fault complex records slip at ~3.8 mm a^−1^, which may be consistent with the published GPS-based current slip-rate estimate of ~6.8 mm a^−1^ across the HFF as a whole.

## Introduction

**Figure 1:**
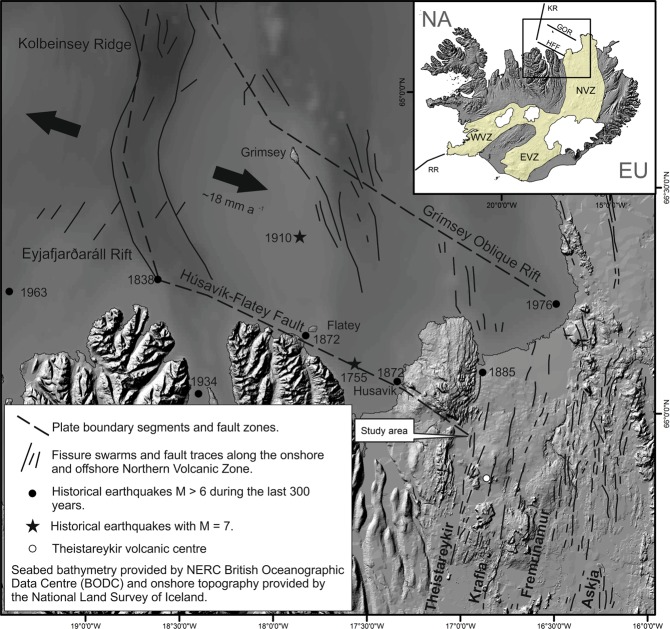
Location map showing the regional context of the study area at the subaerial triple junction formed by the intersection of the Husavik-Flatey Fault (HFF) and the Theistareykir fissure swarm, which marks the western margin of the Northern Volcanic Zone (NVZ). The NVZ forms the European-North American plate boundary in northern Iceland before stepping westwards offshore along the broad Tjornes Shear Zone, predominantly comprising the HFF and the Grimsey Oblique Rift (GOR), and continuing northwards as the Kolbeinsey Ridge (KR). Inset map shows the location in relation to plate-boundary components that cross Iceland, the Reykjanes Ridge (RR), Western Volcanic Zone (WVZ), Eastern Volcanic Zone (EVZ), and the NVZ, HFF, GR and KR. Based on and republished with permission of Blackwell Publishing, from Metzger, S., *et al*. Present kinematics of the Tjörnes Fracture Zone, North Iceland, from campaign and continuous GPS measurements. Geophysical Journal International 192. 441–455 (2012); permission conveyed through Copyright Clearance Center, Inc. Spreading rate for the Kolbeinsey Ridge taken from^4^.

**Figure 2:**
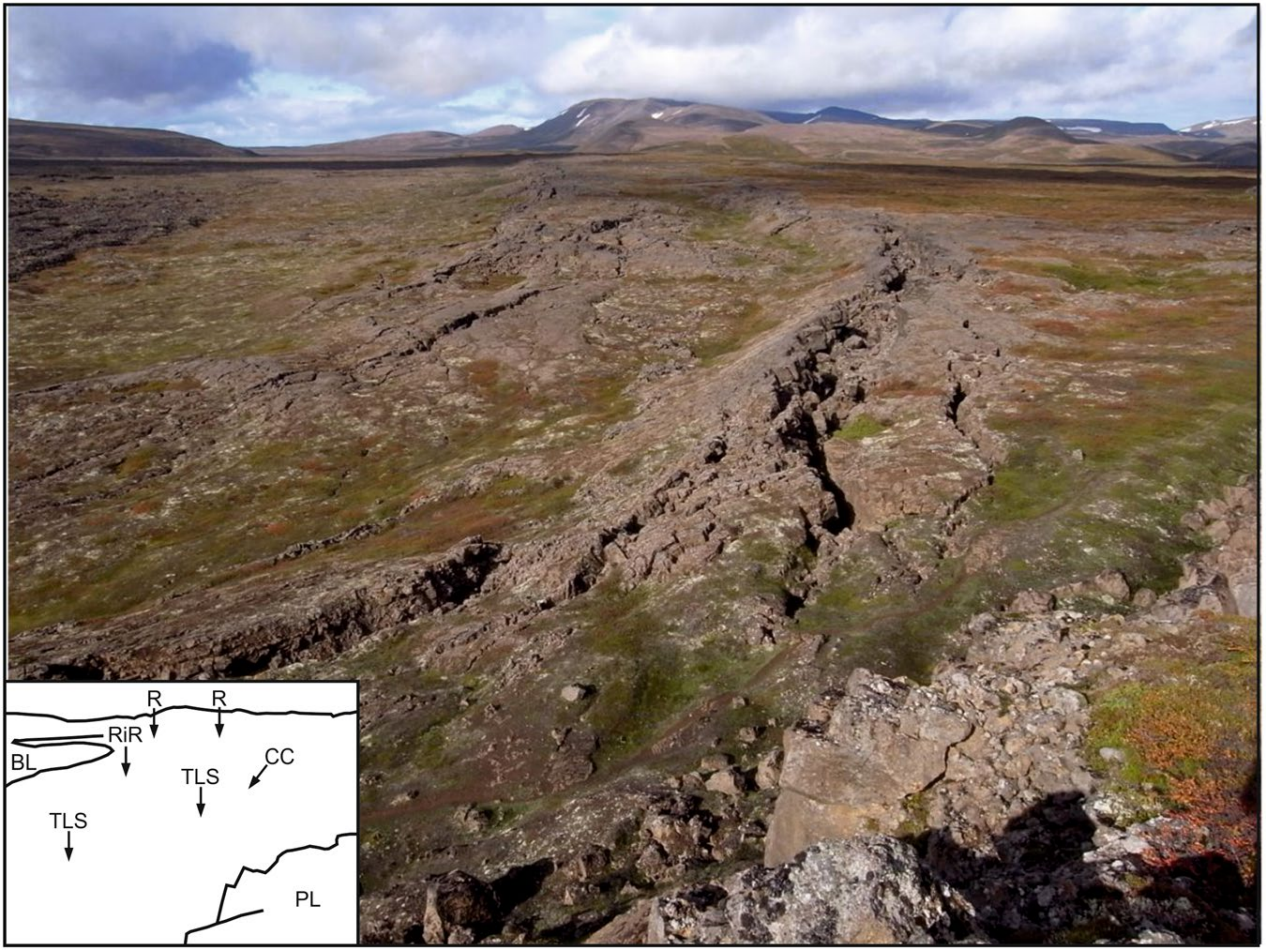
View of the Husavik-Flatey Fault (HFF) zone looking WNW from the main escarpment of the Theistareykir fissure swarm. Inset diagram is designed to aid recognition of key features: BL = blocky lavas; CC = collapsed columns; PL = pahoehoe lavas; R = Riedel fault complex; RiR = Riedel in Riedel faults; TLS = tilted lava slabs. Dominating the view are two left-stepping en-echelon fault complexes, interpreted as first-order R shears, within the HFF. In the middle of the photograph a ridge developed along one of the en-echelon fault complexes is conspicuously transected by a series of left-stepping minor faults; interpreted as Riedel-in-Riedel shears. Note that the prominent faulting in the nearest of the en-echelon fault complexes has opened into a wide fissure as columns within the pervasively columnar-jointed pahoehoe lavas have collapsed inwards under gravity. See text for discussion on the significance of this process. Also note the way in which the dip-slip component of this faulting, down-to-the-left in this view and increasing towards the camera, produces distinctive tilted elongate slabs in the lavas. The faulting is developed in a sparsely vegetated 12.5 ka pahoehoe lava flow field, the surface of which has been separated vertically by several metres along the Theistareykir escarpment. PL on the inset marks this surface in the foreground of the view at the top of the escarpment. Fallen blocks from the escarpment can be seen at the base of the scarp. In the far left middle distance of the view the smooth pahoehoe surface is overlain by the northern edge of blocky 2.4 ka lavas derived from the Theistareykir volcanic centre. See Figs 1 and 3 for map views of these spatial relationships.

Iceland lies astride the European-North American plate boundary, represented in the northern part of the island by the Northern Volcanic Zone (NVZ). Here the NVZ is intersected by the broad Tjornes Shear Zone (TSZ), a dextral transform some 70 km wide that steps the plate boundary 120 km westwards offshore to the Kolbeinsey Ridge, which continues the boundary northwards^1,2^ (Fig. 1). Global plate motion models and GPS results^3–6^ are in good agreement and suggest current plate motion across the TSZ is ~18 mm a^−1^. The principal onshore component of the TSZ, the Husavik-Flatey Fault (HFF), with a GPS-based current slip rate estimate of ~6.8 mm a^−1^, has an on-land extent of some 25 km that displays classic features of active strike-slip tectonics before intersecting the western margin of the NVZ, here marked by a belt of normal faulting and eruptive fissures known as the Theistareykir fissure swarm, to produce a remarkable subaerial exposure of ridge-transform related triple junction deformation features^2,7,8^ (Figs 1 and 2) normally obscured within the oceanic realm^9–11^.

Four major earthquakes^6^, estimated at M6.5 or greater, have occurred on the HFF since 1755 (Fig. 1). The most recent event, in 1872, caused extensive damage and surface faulting in and around the town of Husavik. Vertical offsets on the HFF amount to some 200 m near Husavik, and may reach 1400 m in total^12^, a figure consistent with a vertical displacement estimate of 1100 m off the coast at the island of Flatey^13^ (Fig. 1). Estimates of total horizontal offset vary: Saemundsson^14^ suggests 5–10 km, Young *et al*.^15^ suggest 20 km, and Gudmundsson^16^ suggests a total right-lateral offset of ~60 km. Initiation of the HFF is estimated to have occurred 7–9 Ma ago^1,16^. For the NVZ the most notable historical activity is the 1975-84 Krafla eruption south of the study area, which was accompanied by episodic rifting deformation amounting to several metres horizontally, and a few metres vertically, as well as by numerous M5.0–6.5 earthquakes^5,17–20^.

**Figure 3:**
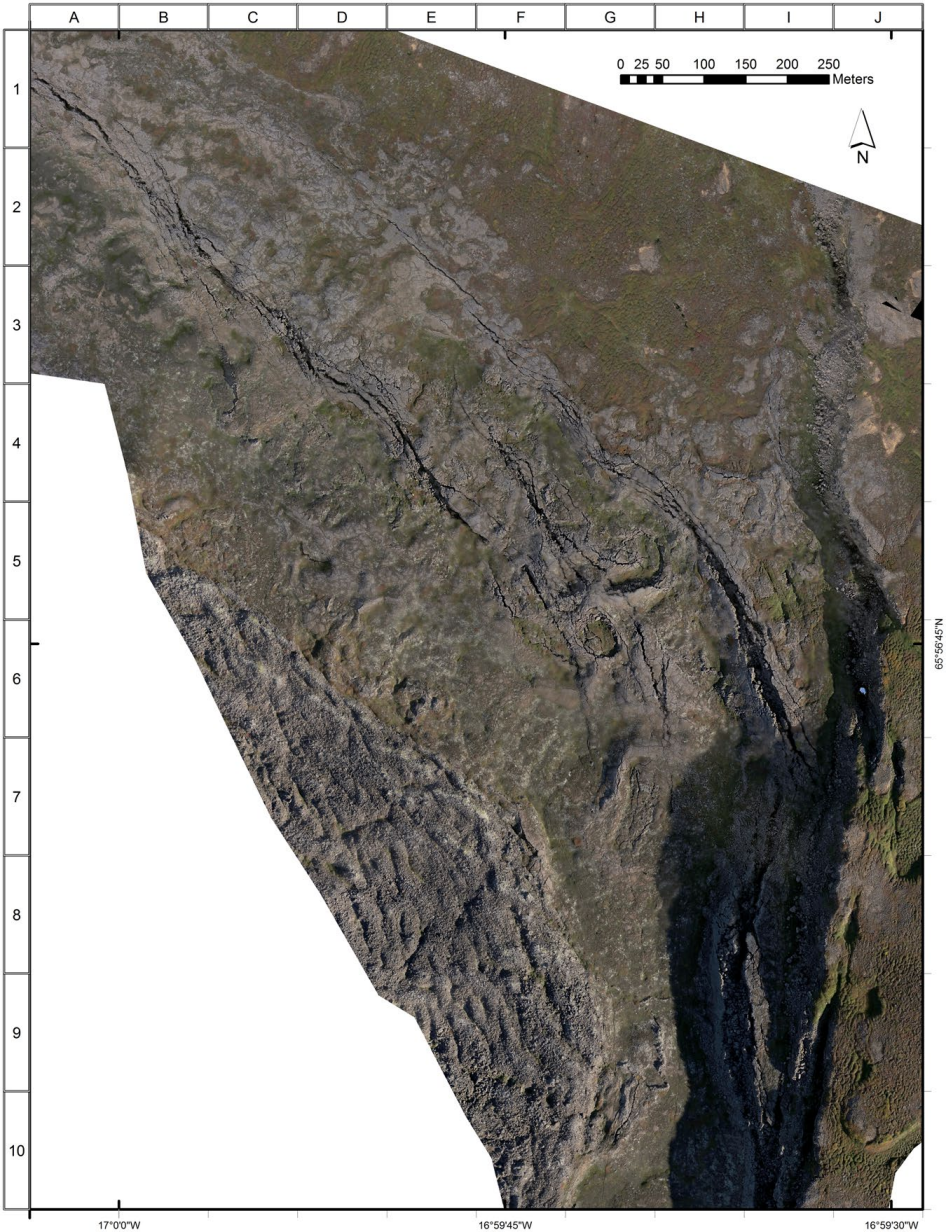
Low-sun drone imagery of the triple junction showing the WNW-striking Husavik-Flatey Fault intersecting the N-S Theistareykir rifting. Note the contrast between the smooth surface of the pahoehoe flow field, in which the triple-junction structures are developed, and the younger blocky lavas occupying the south-eastern part of the image. See also mapping in Fig. 4.

Mapping in the wider study area documents the very complex regional interaction between rifting and transform faulting^21–25^. The deformation structures are developed in an extensive sheet of pahoehoe lava flows, constrained to about 12.5 ka BP^26,27^, emitted from the Theistareykir central lava shield to the south of the study area (Figs 1 and 2). Notably, these lavas form a smooth and planar *tabula rasa* that provides a uniquely preserved long-term record of subsequent structural interactions. More recently the 2.4 ka ‘Theistareykjhraun’ lavas, easily distinguished by their blocky character, advanced from the shield northwards, and were constrained to the SE corner of the study area by tectonic uplift at the triple junction (Figs 2 and 3). These fortuitous circumstances, coupled with only minor vegetation development, create an area of ~1 km^2^ containing innumerable fault features displayed in exquisite detail. From the ground, features such as piercing points, slip directions and amounts, and other structural features can be recognised and measured very accurately. A survey on foot also enables primary lava features, such as fracturing associated with inflation and deflation in the pahoehoe flow field, to be recognised. However, crucially, distinguishing between these primary fractures and the subsequent innumerable tectonic fractures is often extremely difficult at ground level. Summarising: the overwhelming wealth of detail, some potentially misleading, demands a bird’s eye perspective and to be assembled and analysed in a map view, an essentially impossible task using conventional base maps or aerial photographs.

### Methodology

**Figure 4:**
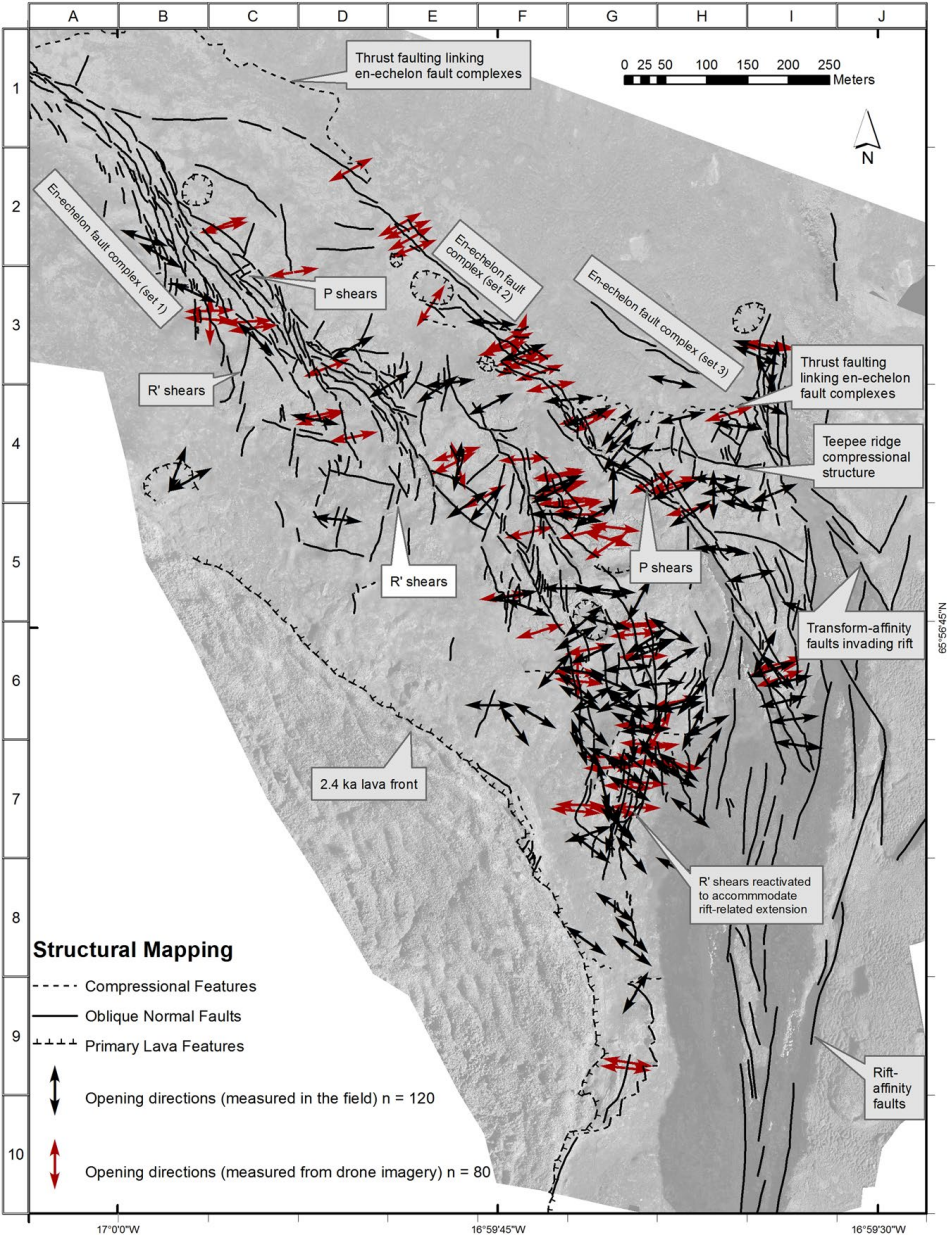
Map of faulting, fault slip opening directions (black = measured in the field, red = measured from the imagery), and primary lava features. The annotations, and the alpha-numeric grid on the western and northern borders of the map, are designed to aid identification of features discussed in the text.

**Figure 5:**
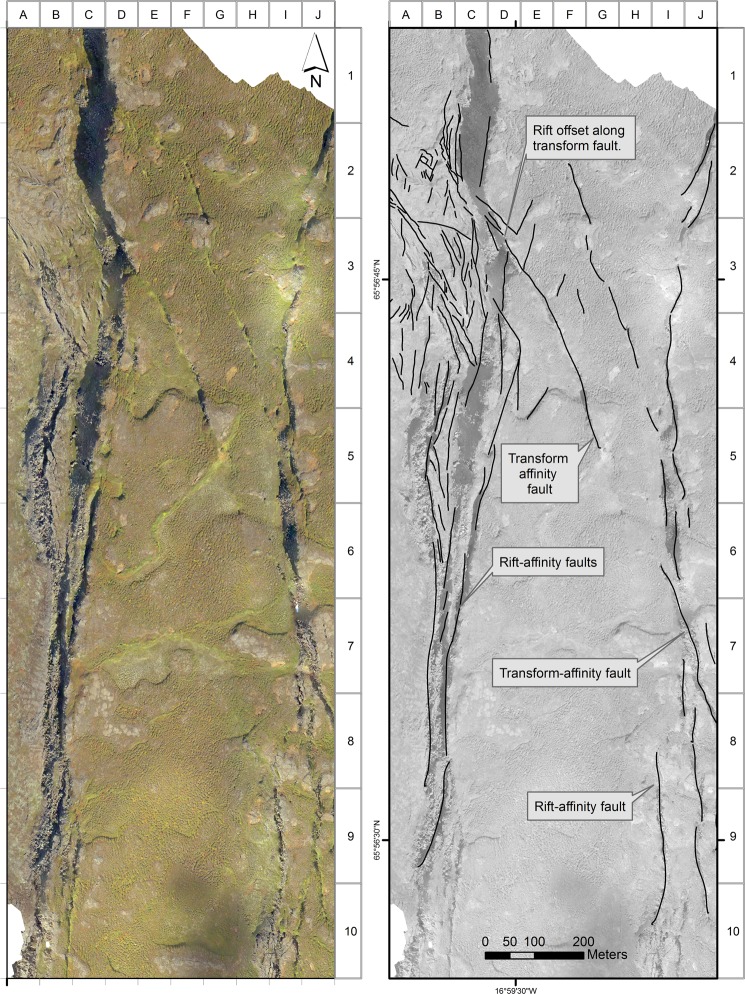
High-sun drone imagery showing the Theistareykir rifting, in shadow on Fig. 3, and the eastward encroachment of HFF transform-affinity faulting. See text for discussion.

To address the need for very large-scale high-resolution imagery we coupled detailed GPS-referenced ground measurements and field surveys with mapping at up to 1:200 scale using imagery obtained from low-altitude GPS-controlled drone surveys (Fig. 3), creating for the first time a large-scale map that fully documents this unique site (Figs 4 and 5). Two separate drone surveys were undertaken, both in cloud-free conditions. The first survey exploited low-sun angle conditions to best display subtle features in the study area, while the high-sun survey conditions during the second drone mission helped reveal detail in areas of deep shadow.

Both surveys used an Unmanned Aerial Vehicle-Structure from Motion (UAV-SfM) framework, within which aerial photography was acquired using a small multi-copter aerial unmanned vehicle and then processed to generate an ortho-rectified image mosaic and digital surface model (DSM). The UAV used was a 3D Robotics IRIS quad-copter fitted with onboard autopilot and GPS receiver, capable of automatic mission planning using the open source Mission Planner software that allows missions to be pre-planned and then uploaded to the autopilot prior to take-off. A camera mounted beneath the drone captured vertical overlapping photographs of the ground surface at a resolution of 12 megapixels. Six GPS ground control points were installed across the study area using white markers to provide GPS calibration for post processing of the photography, and a flight plan was constructed using a shapefile boundary area to calculate a set of GPS way points for the take-off location, flight path, turning points and landing location. The camera was programmed to trigger every three seconds, ensuring overlapping photographs were acquired to cover the study area in stereo. A flying height of 70–90 m was chosen to produce imagery with a ground separation distance (GSD) of approximately 4 cm, and the imagery was processed using the commercial software package Agisoft PhotoScan version 1.4. The technical background of this approach is not detailed here, but UAV-SfM for geological applications has been described by Bemis *et al*.^28^, Carrivick *et al*.^29^ and Smith *et al*.^30^.

Field measurements of fault slip directions and opening amounts were obtained using a folding rule, a compass and a hand-held GPS. Working systematically across the study area produced some 120 measurements, up to a maximum slip of about 1 m. At these short distances the plunge of the slip directions was too small, <10 degrees, to be measured with meaningful accuracy in the field and was disregarded. Mapping from the imagery was carried out on a workstation using ArcGIS software at scales ranging from 1:5000 to 1:200, depending on the size and continuity of the features being mapped. The larger end of that scale range was useful for very small features on the ground, while still retaining the crucial bird’s eye perspective. Overall, the scale that proved most valuable in the mapping was 1:500.

## Results

The combination of over 120 rigorously controlled ground measurements and high-resolution geo-referenced drone imagery makes possible an extremely detailed map of this remarkable area, recording a very dense pattern of faults (>200), kinematic data and primary lava features (Figs 3–5). These results are presented below under four sub-headings, with the letter/number co-ordinate system around the margins of the maps in Figs 4 and 5 used to locate the features described. Annotations identifying key features have also been added to these figures. Note that, in order to present the results under one heading as clearly as possible, some interpretation is also included. In all cases this amplification of the results is clearly identified as interpretation.

### Transform-rift interactions

Overall, the two fault systems, the dominantly NW-SE transform-affinity faults of the HFF and the dominantly N-S down-to-the-west normal faults of the Theistareykir rifting, can be clearly distinguished (Figs 3 and 4). In the field the western margin of the Theistareykir rifting is represented by a conspicuous and essentially continuous fault scarp that extends north and south well beyond the triple junction itself; being generally higher, up to several metres, and better defined, to the south. Throughout this wider area the scarp is developed solely in the ~12 ka pahoehoe lavas (Fig. 2). To the east, inboard of this rift front scarp, there are numerous typically smaller N-S rift-affinity fault scarps, but these are absent to the west (Fig. 1). The HFF can be traced in the field as a series of fault scarps extending inland from the coast but, on reaching the ~12 ka lavas, becomes confined to a distinctive belt of faulting that displays right lateral movement with very limited scarp development (Fig. 2).

Within the area of detailed study (Figs 3–5), both fault systems become broader and more complex, suggesting structural interactions. Starting only about 0.5 km from the rift front scarp the HFF exhibits a marked and progressive increase in the extensional component of slip (Fig. 2), ultimately becoming strongly extensional and apparently becoming reactivated to accommodate rift-related normal faulting. This pattern is confirmed by measured opening directions showing dominant extension (I6 in Fig. 4), and by intact linear rafts of pahoehoe lava now dipping steeply westwards between individual fault strands (Fig. 2). These observations are consistent with the notion that the rift front acts as what might be envisaged as a bounding wall to the faulting, with the right-lateral transform-affinity faulting operating in a way analogous to the margin of a pull-apart basin, transporting material generally westwards on the south side of the transform.

In strong contrast, on the opposing north side of the transform, right-lateral offset produces compression, with transform-affinity faulting conspicuously invading eastwards beyond the rift front before curving to a more N-S strike and apparently being subsumed within the extensional faulting of the rift zone (C to J, and 2 to 8, in Fig. 5). Another possible manifestation of this invasion occurs where the northernmost transform-affinity faulting cuts the main rift front escarpment at the northern edge of the triple junction (C-D, 2–3, in Fig. 5). Here the escarpment displays a seemingly-inconsistent left-lateral inflection, which may possibly be interpreted as due to a fault-bounded wedge being carried right-laterally immediately north of the transform. However, the overall strike of the rift front is maintained without lateral displacement north and south of the triple junction, with interactions between the two systems being restricted to ~800 metres of the rift front (I-J, 5–9, in Fig. 4).

**Figure 6:**
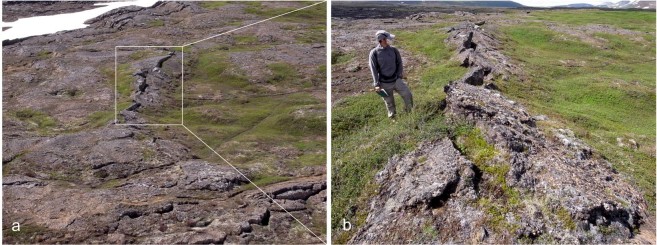
Field photographs of compressional teepee ridge. (**a**) View westwards from the Theistareykir escarpment showing the ridge developed in pahoehoe lavas. The linear snowbank and visible faulting in the NW corner of the view mark the Husavik-Flatey Fault as it approaches the escarpment. (**b**) Along-strike view of the teepee ridge looking west. See H-4 in Fig. 4 for location.

On the opposing, western, side of the rift compression north of the transform appears to have produced an E-W-striking linear fault over 100 m in length (H4 in Fig. 4), with a contractional component of movement producing a conspicuous teepee ridge in the pahoehoe surface that documents shortening of ~1.5 m (Fig. 6.) Further consideration of this structure is provided under the sub-heading below.

### Transform-affinity tectonics

The drone imagery and field measurements of opening directions indicate that the HFF zone displays classic structural features associated with right-lateral simple-shear tectonics. Notwithstanding that the *sensu stricto* requirement for constant volume during simple-shear deformation is unlikely to be met. Mapping reveals two conspicuous left-stepping en echelon fault complexes within the transform zone (Fig. 2), with a less pronounced third left-stepping en echelon fault complex recognisable as it extends eastwards into the N-S rift front (Fig. 4). These three regional scale structures, made up of numerous faults, many of which display right-oblique slip documented by field measurements, are interpreted as first-order Riedel shears. Their sense of stepping is consistent with overall right-lateral displacement on the HFF.

The westernmost of these three main en echelon fault complexes displays three repeated sets of closely spaced left-stepping right-oblique faults (Fig. 2; B-C 2–3, D3 and E4 in Fig. 4), interpreted to be second-order Riedel shears and thereby constituting a Riedel-in-Riedel arrangement. At least one similar set of subsidiary shears, again interpreted as a Riedel-in-Riedel development, occurs along the second main en echelon fault complex to the east (G4 in Fig. 4). Further east, at H4 in Fig. 4, this pattern may be repeated by a set of minor faults intersecting the northern side of the same fault complex, although more complete development here may be inhibited by proximity to the rift front.

These en echelon sets of second-order faults, interpreted as Riedel-in-Riedel features, are linked by a further set of faults made distinct, firstly, by a strike closer to E-W, anticlockwise from the overall strike of the en echelon fault complexes, second, by their lack of consistent sense of stepping and, thirdly, where measured in the field, by their synthetic offset sense with respect to the transform (C3, D3, E4 and G4 in Fig. 4). Based on the above observations these linking fault sets may represent P-shears, an interpretation also consistent with the long duration of simple-shear deformation recorded by the ~12 ka pahoehoe lava field^31,32^.

Further evidence for a long history of simple shear is provided by a discontinuous set of minor faults over a broad belt at a high angle to the main en echelon fault complexes, notably the westernmost of the three identified. These are best expressed in grid squares D-E, 4–5 in Fig. 4. Notably, where opening directions could be measured on these faults, the sense of offset is left oblique. This, together with the fault orientation, suggests these structures formed as R’ shears, and may originally have been more continuous. Sets of right-stepping minor fractures straddling these R’ shears can be recognised in places, for example at D4 in Fig. 4, and these are interpreted as a R’ Riedel-in-Riedel structural response to localised R’ left-lateral displacement. If this interpretation is correct, this represents, to our knowledge, a structural development not previously recognised in the field; possibly again attributable to the long record of deformation within the lava sheet.

Additionally, the R’ orientation is sub-parallel to the main Theystareykir rifting and it seems likely that, as the rift is approached, original R’ shears have assumed a role in accommodating rift extension, with measured pure dip-slip opening directions. Examples supporting this suggestion occur particularly at the SE end of the westernmost of the three main en echelon fault complexes, in grid squares G-H, 6–7 in Fig. 4.

**Figure 7:**
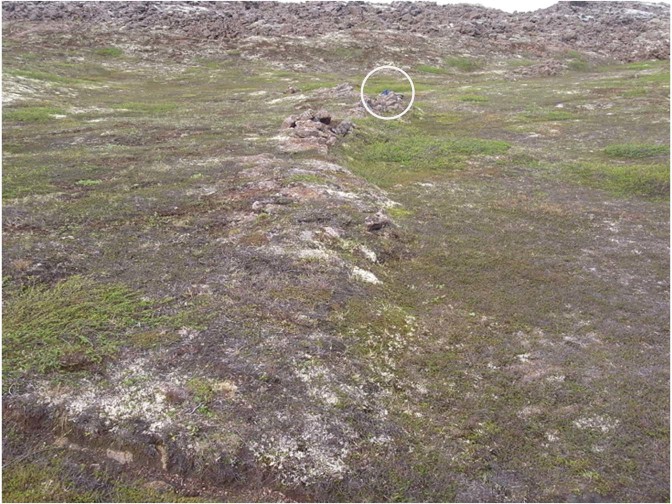
Field photo of thrust linking two en echelon shears within the pahoehoe lavas. White circle highlights a small blue rucksack for scale. The younger blocky lavas are conspicuous in the background. The direction of tectonic transport is from left to right, and vertical separation is ~1 m.

Low angle compressional structures are also evident within the HFF transform zone, both on the ground and in the imagery (Fig. 7). These structures displace the pahoehoe lava field surface vertically by approximately a metre and link en echelon faults, most conspicuously the three main left-stepping fault complexes described above. They can be distinguished in the mapping by their position, and by their irregular arcuate surface trace, which contrasts to the linear steeply-dipping faults that predominate in the triple junction (A-D1 and G-H3–4, Fig. 4). They are interpreted as the surface expression of compressional strike-slip relay ramps and, once again, are thought to reflect the long history of simple-shear tectonics recorded at this site^33^.

The linear teepee ridge structure (Fig. 6), described under the previous sub-heading, occurs a short distance south of the easternmost of the above low-angle faults, and also links two of the left-stepping en-echelon fault complexes (G-H4, Fig. 4). This structural association is repeated between the NW end of the central of the three main en echelon complexes and the left-stepping westernmost complex. Here a prominent curvilinear fault, generally E-W-striking, links the NW end of the central en echelon complex. This can be seen to the south of the irregular surface trace of the low-angle compressional fault described above (B-D1, Fig. 4). This curvilinear fault has also produced a linear ridge, although lacking the teepee feature, and may represent a transpressional linkage, which, if interpreted correctly, cuts down to the relay ramp that daylights to the north. This structural repetition suggests a common origin at these two compressional transfer zones in the transform, with the teepee ridge possibly reflecting increasing compression as the triple junction is approached.

**Figure 8:**
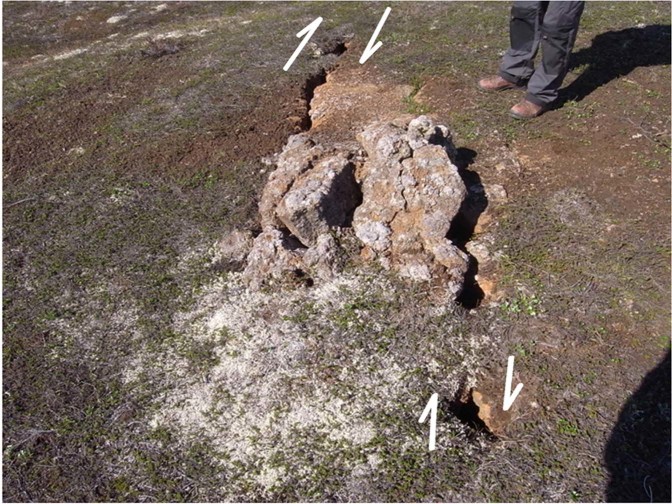
Beautifully preserved compressional deformation within a small transfer zone between two minor left-stepping right-lateral en-echelon faults within the HFF.

Finally, in documenting the structures at this remarkable site, Fig. 8 shows a delightful compressional transfer zone and associated push-up structure between two minor left-stepping en-echelon faults within the transform. This gem occurs at F5 in Fig. 4.

### Primary lava features and long-term slip rates

In carrying out the fieldwork it became obvious that some of the deformation and fracture development in the study area was not tectonic, but represented primary features associated with emplacement of the lavas; most notably these features included inflation-deflation effects within the pahoehoe flows. The bird’s eye perspective afforded by the drone proved invaluable in distinguishing and mapping these primary features (Figs 3 and 4).

**Figure 9:**
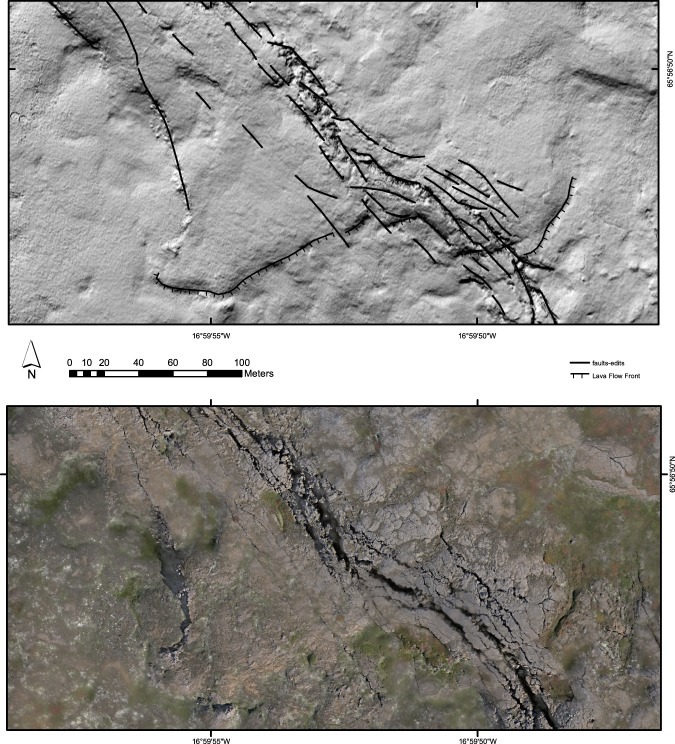
Obliquely illuminated and low-sun drone images of a flow front within the pahoehoe lava field offset along one of the en-echelon fault complexes, interpreted as first-order R shears, within the HFF zone of faulting. Offset is ~48 m. Note the open fissures developed along the more prominent faults in the low-sun view by columnar jointing-controlled gravity-induced collapse. See text for discussion.

As shown in Fig. 9 the primary features included a prominent pahoehoe flow front within the lava field that records a total cumulative offset on one of the en echelon fault complexes within the transform of ~48 m. This offset of the 12.5 ka flows indicates a post-glacial average slip rate of ~3.8 mm a^−1^.

### Field measurements, and measurements from drone imagery

**Figure 10:**
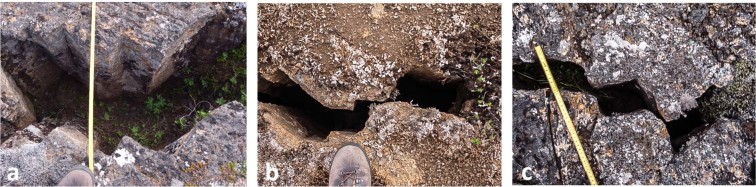
Field photographs showing examples of, (**a**) pure extension, (**b**) right-oblique and, (**c**) left-oblique faulting. Note the matching irregular walls of the faults, imparted by columnar jointing in the lavas, which enable piercing points and opening amounts to be accurately identified and measured. With opening amounts greater than about 1 metre inward collapse of columns creates open fissures along the faults and obliterates piercing points.

Systematic and painstaking examination of the study area on foot identified some 120 opportunities to confidently and accurately measure the trend and amount of fault displacement between piercing points, as shown in Fig. 4. Figure 10 illustrates field examples of piercing points that record pure extension, left-oblique and right oblique extensional movement on faults within the transform. All 120 measurements were for offsets of less than one metre; beyond that distance reliable field measurements could not be made because of modification of the opposing walls of faults by localised gravity-driven collapse. Collapse exploited columnar jointing that is pervasive throughout the lava sheet, so that individual columns, typically 10–20 cm in diameter, become detached, obscuring piercing points and exaggerating opening amounts. This process invariably results in faults developing into large open fissures containing a jumble of numerous fallen columns (Figs 2, 3 and 9).

While recognising this problem it was nevertheless decided to take the opportunity afforded by the drone imagery to supplement the field data with remotely sensed measurements. Eighty opening direction measurements were made from the imagery and these are included, differentiated by colour, in Fig. 4. The two sets of measurements are discussed in the following section.

## Discussion

The results presented above, along with specific highlighted interpretations, demonstrate the uniqueness and significance of this remarkable site. Most notably this arises from the ‘clean sheet’ represented by the smooth pahoehoe lava flow field that, fortuitously, resurfaced the triple junction area at the end of the last glacial period, providing in exquisite detail a ~12 ka record of subsequent faulting, which is ongoing in a structural setting that is fundamental to global tectonics, but normally unavailable for study. The pahoehoe lavas represent an ideal material for preserving this long record of deformation, which is thought to include such features as P shears, compressional strike-slip relay ramps and Riedel-in-Riedel relationships that require multiple faulting events to develop and become recognisable. The detailed record of these simple-shear-affinity structures is notably absent further west, towards the coast, beyond the pahoehoe outcrop area; an observation consistent with the relative youthfulness of the pahoehoe flow field. This is likely to favour preservation of minor and early-formed Riedel structures that tend to become less active as through-going PDZ (Principal Displacement Zone) structures become established as the fault zone matures through multiple faulting events^34^, although undoubtedly variations in erodibility of the volcanic bedrock along the HFF also plays a role. By comparison, the classic^35^ study of simple-shear deformation, although extremely detailed, documents the effects of a single faulting event, the 1968 Dasht-e-Bayez earthquake in Iran. Moreover, the deformation was recorded in unconsolidated playa lake deposits and quickly erased. Additionally, the ~12 ka record under discussion represents a natural laboratory, unlike the accelerated model conditions represented by comparable clay-cake studies such as the landmark work of Wilcox *et al*.^36^ and more recent similar studies reviewed in Dooley & Schreurs^37^. These geological attributes may also favour the R’ Riedel-in-Riedel structural record, thought to be previously unrecognised and, overall, the repetition of structures across a range of scales suggests fractal relationships may be recorded.

The exceptional quality of the exposure afforded by the pahoehoe sheet demonstrates that rift-affinity and transform-affinity fault interactions at the triple-junction, over the past ~12 ka, are restricted to a small area, and that the position of the triple-junction itself has remained remarkably constant. As highlighted in the results section, above, the prominent fault scarp marking the western margin of Theistareykir rifting is very continuous and well developed. Given its tectonic setting, and the size of typical historically and instrumentally recorded earthquakes, we consider it represents construction by numerous faulting events recurring on essentially the same fault. The outcrop pattern of the blocky lavas, including their absence from the upthrown side of this fault in the wider study area, demonstrates that the scarp was well established 2.4 ka-ago, and has not migrated westwards (Figs 2 and 3). It is conceivable that some development of this faulting westwards is concealed by the blocky lavas, but these are thin and any significant interconnected normal faulting would be recognisable. Similar arguments based on the blocky lava outcrop pattern can be made for the position of the triple-junction intersection between this fault and the HFF. Undoubtedly, over the last ~12 ka, some Theistareykir rifting has been accommodated on scarp-forming faults further east. However, the lack of a comparably prominent and continuous fault scarp in the vicinity suggests the western margin of Theistareykir rifting, at least for the past ~12 ka, has not migrated to the west.

This interpretation is also consistent with faulting relationships mapped and already described, above, inboard of the triple junction itself. Here, only limited areal encroachment of transform-affinity faulting is observed, before it curves into alignment with the rift and loses its identity. Moreover, if the present position of the western margin of Theistareykir rifting had stepped westwards in the past ~12 ka, the lavas inboard of the present position of the triple junction should preserve a complex record of interactions created during the existence of a more easterly former position for this junction. On the opposing, western, side of the Theistareykir margin there is evidence for only very limited, approximately 300 metres, westward encroachment of rift-affinity displacement. As outlined in Results this is restricted to immediately south of the triple junction, where localised extension can be expected from right-lateral transform-related movement. This encroachment is interpreted to represent reactivation of favourably aligned faults that began as R’ shears in the transform, and original R shears progressively realigned as they approach the margin of rifting (G-I, 6–7, Fig. 4). Similarly, if the triple junction itself had migrated southwards along the rift margin during the past ~12 ka, some evidence of now-inactive HFF faulting would be expected to be preserved in its wake to document this. Ruling out migration of the junction in the opposite direction, to the north, is made less easy by the covering of blocky lavas, but there is no field evidence to suggest these obscure a former position.

Detailed documentation and analysis of the triple-junction itself, restricted both temporally and spatially, remains the aim of our work. We do not suggest relationships in this small area are representative of wider NVZ-TSZ interactions, which have evolved across a broad region over some 8.5 Ma and include the locus of activity migrating northwards over time^2^. Triple-junction evolution and fault interactions over this much greater temporal and spatial framework, including triple-junction stability^38,39^, are outside the scope of the present paper. Several recent papers have considered interactions between the HFF and the NVZ at a more regional scale over a more extended time period^23,25,40,41^.

The post-glacial average slip rate of ~3.8 mm a^−1^ recorded on the westernmost of the three en echelon fault complexes, which are interpreted as first order Riedel shears within the transform, may be compared with the GPS-measured estimate of ~6.8 mm a^−1^ for the HFF. The disparity of ~3.0 mm a^−1^ may reflect transform displacement accommodated by other structures, identified above, across this wide zone of active faulting as the HFF approaches the Theistareykir rifting (Fig. 3). Another possible factor in the disparity is variation in slip rate through time, particularly in view of the very short GPS record in comparison to the ~12 ka geological record. Further discussion of possible explanations for this disparity, which has important implications for seismic hazard assessment for the HFF and other seismogenic structures, can be found in Rust *et al*.^42^.

The detailed bird’s eye view offered by the drone imagery proved invaluable for mapping the detailed spatial relationships of the tectonic deformation and, crucially, for distinguishing them from primary features, particularly inflation-deflation effects, associated with emplacement of the lavas. At ground level these features were typically impossible to reliably differentiate from small nearby faults. Nevertheless, while embracing the drone platform as an exciting tool in such detailed studies, a couple of points may be usefully highlighted. Firstly, our experience confirms the value of low-sun conditions for retrieving the maximum detail from generally low-relief terrains such as this lava field. This had the drawback of deep shadows cast by the main Theistareykir escarpment, so our second drone survey sought to address this problem by flying when the sun was higher. Although this alleviated the shadow problem it reduced definition of smaller features and, unexpectedly, the relative lack of clear linears in the high-sun imagery appeared to hamper the software in accurately stitching together some of the individual images taken from the drone. Second, the 80 measurements of opening directions and amounts taken from the drone imagery proved to be significantly lower in quality and reliability in comparison to the 120 field measurements. The pervasive columnar jointing in the lavas provide discontinuities along which gravity-induced collapse occurs, destroying piercing points and exaggerating opening amounts. Consequently, as shown by comparing directions from the two datasets in Fig. 4, measurements from the drone imagery typically missed the strike-slip component of fault movement and misleadingly emphasised pure extension.
